# The spatiotemporal dynamics of spatially variable genes in developing mouse brain revealed by a novel computational scheme

**DOI:** 10.1038/s41420-023-01569-w

**Published:** 2023-07-27

**Authors:** Yingzhou Hong, Kai Song, Zongbo Zhang, Yuxia Deng, Xue Zhang, Jinqian Zhao, Jun Jiang, Qing Zhang, Chunming Guo, Cheng Peng

**Affiliations:** Center for Life Sciences, School of Life Sciences, Yunnan University, Kunming, 650500 China

**Keywords:** Computational neuroscience, Development of the nervous system

## Abstract

To understand how brain regions form and work, it is important to explore the spatially variable genes (SVGs) enriched in specific brain regions during development. Spatial transcriptomics techniques provide opportunity to select SVGs in the high-throughput way. However, previous methods neglected the ranking order and combinatorial effect of SVGs, making them difficult to automatically select the high-priority SVGs from spatial transcriptomics data. Here, we proposed a novel computational pipeline, called SVGbit, to rank the individual and combinatorial SVGs for marker selection in various brain regions, which was tested in different kinds of public datasets for both human and mouse brains. We then generated the spatial transcriptomics and immunohistochemistry data from mouse brain at critical embryonic and neonatal stages. The results show that our ranking and clustering scheme captures the key SVGs which coincide with known anatomic regions in the developing mouse brain. More importantly, SVGbit can facilitate the identification of multiple gene combination sets in different brain regions. We identified three dynamical sub-regions which can be segregated by the staining of Sox2 and Calb2 in thalamus, and we also found that Nr4a2 expression gradually segregates the neocortex and hippocampus during the development. In summary, our work not only reveals the spatiotemporal dynamics of individual and combinatorial SVGs in developing mouse brain, but also provides a novel computational pipeline to facilitate the selection of marker genes from spatial transcriptomics data.

## Introduction

Since the brain develops in a series of spatially organized events with highly diversified neuronal sub-populations [1], it is important to identify the spatially variable genes (SVGs), referring to the genes which are not randomly but specifically expressed in one or several regions in the tissue, to delineate the brain sub-regions and cell subtypes during the development. The rapid development in spatial transcriptomics provides huge opportunities to select novel SVGs in the high-throughput way [2, 3]. One recent work utilized the spatial transcriptomics to define brain regions for adult mouse but without clearly showing the marker genes [4]. Another work investigated the mouse cerebral cortex by integrating single-cell RNA sequencing (scRNA-seq) and spatial transcriptomics [5]. Researchers also used the spatial transcriptomics to investigate the gene expression distributions in certain brain regions [6–8]. However, many brain regions are still less studied in spatial transcriptomics, so it is still important to systematically explore the marker genes in high-throughput way for the anatomic regions and sub-regions in the mouse brain.

One basic computational task in spatial transcriptomics data is to detect SVGs for biological applications. Recently, some unsupervised methods were proposed to identify SVGs from spatial transcriptomics data without histological images, such as Trendsceek [9], SpatialDE [10], Spark [11], and SOMDE [12]. However, these works neglected the ranking effect of SVGs, especially their combinations, in marking the anatomic sub-regions and cell subtypes. The methods to cluster spatial domains were also proposed [13–17], but the similarity clustering cannot satisfy all cases, especially the researchers who frequently use combinatorial genes to mark tissue sub-regions and cell sub-types. Compared to spatial domain clustering, combinatorial genes also show obvious advantages: flexibly defining more diversified tissue sub-regions/nuclei as well as cell subtypes, even the subtle ones marked by one or two important genes, with the user’s own biological purpose. However, it is difficult to manually select the gene combinations from the huge space in high-throughput spatial transcriptomics dataset. Thus, it is critically important to develop new methods to rank the individual and combinatorial SVGs to facilitate marker selection.

In this work, we developed a novel computational pipeline SVGbit to effectively rank the individual and combinatorial SVGs to select the potential marker genes for tissue regions and sub-regions. We tested the ranking effect in publicly available spatial transcriptomics datasets for both human and mouse brains. Since there is no other work to explore the marker effect of combinatorial SVGs, we then investigated the developing mouse brain by generating spatial transcriptomics and immunohistochemistry (IHC) data at critical embryonic and neonatal stages. By applying SVGbit to the generated data, we identified important SVGs and their combinations which could provide novel insights on the spatiotemporal development for mouse brain. As we know, our work is the first one to systematically rank the individual and combinatorial SVGs to facilitate the marker selection for various brain regions.

## Results

### Computational pipeline and evaluation

**Fig. 1:**
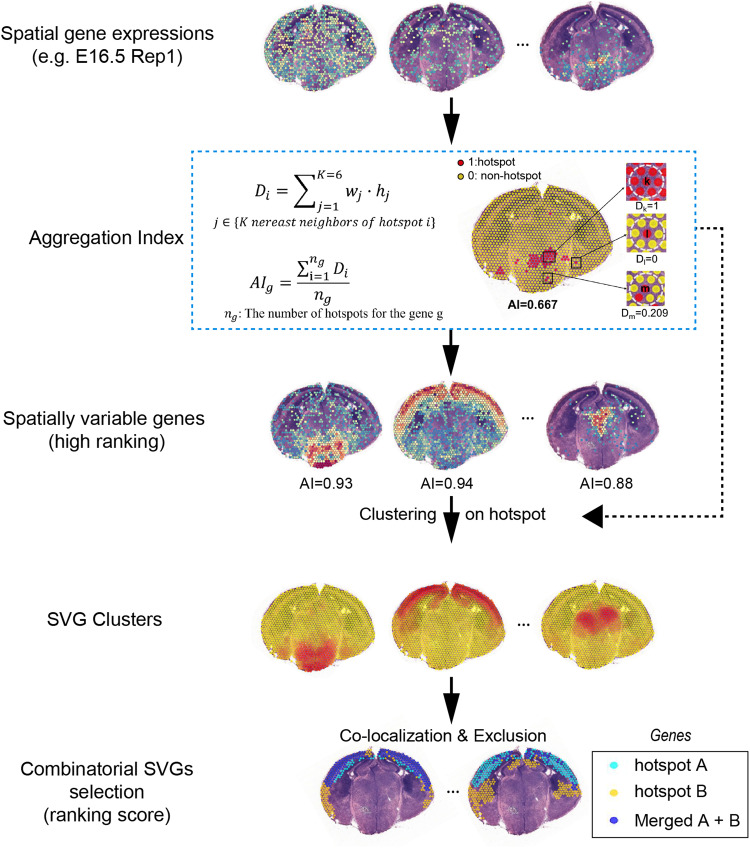
The schematics of computational pipeline. SVGbit mainly consists of four modules, including aggregation index calculation, individual high-ranking SVG selection, SVG clustering and scoring for combinatorial SVGs. In the first and third rows, the color from blue to red represents spatial gene expressions from 0 to high value. The color from yellow to red represents the hotspot ratio from 0 to the high value in the SVG clusters.

In our practice, many SVGs showed complex spatial relationship which could provide biological insights or applications (Supplementary Fig. 1), but these combinatorial effects were neglected by previous methods [9–12]. We also found that previous SVG detection methods [10–12] had little ability to distinguish top SVGs since lots of *P*-values showed the same small values (Supplementary Tables 1 and 2), making them inconvenient for automatic marker selection. Inspired by these observations, we developed a novel computational pipeline, called SVGbit, to rank the individual and combinatorial SVGs (Fig. 1 and Supplementary Fig. 2). First, the spatial gene expressions are used to calculate the hotspots for each gene. Second, the local hotspot density is calculated to take the neighbor hotspots into consideration. And the aggregation index is defined as the averaged local hotspot density to represent the overall spatial aggregation of all hotspots. Third, the high-ranking SVGs are clustered to reduce the number of combinatorial SVGs since we can mainly focus on the SVGs in the same and overlapped clusters. Finally, the combinatorial SVGs in the selected clusters are scored according to their spatial co-localization and exclusion (details see “Materials and methods”).

**Fig. 2:**
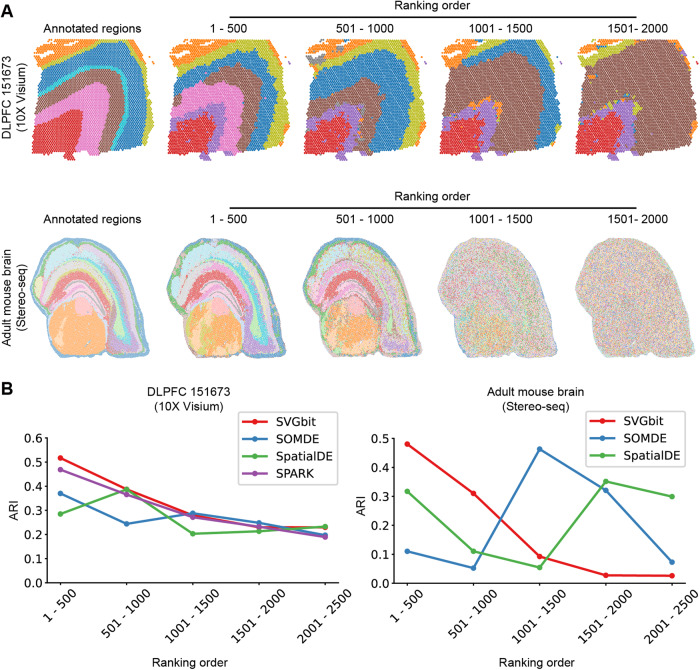
Evaluation on the SVGbit. **A** The spatial domains along with ranking order. **B** The comparisons among different SVG detection methods. The x-axis and y-axis represent ranking order and adjusted rand index (ARI), respectively.

We first evaluated the ranking effect of aggregation index on individual SVGs by using the publicly annotated datasets, the mouse brain generated from Stereo-seq at around cellular resolution [18] and the human dorsolateral prefrontal cortex generated from 10X Visium at relatively lower resolution [19]. The top SVGs were divided into groups in the descendant ranking order, and the BayesSpace [13] was used to identify spatial domains by using the grouped SVGs. As shown in Fig. 2A, the spatial domains derived from top 500 SVGs capture the main characteristics of the annotated tissue regions in both high-resolution and relatively low-resolution datasets. Though the SVGs with lower ranking order still capture some characteristics of anatomic regions, the spatial domains gradually become messy and even disappear along with the descendant ranking order.

We also compared SVGbit to other methods which detect SVGs without histological images. The softwares SPARK, SpatialDE, and SOMDE were run by using the parameters recommended in the online documents (Supplementary Tables 3 and 4), and the *P*-values or *Q*-values reported in these methods were used to rank the detected SVGs. The adjusted rand index (ARI) [20] was used to measure the similarities between clustered domains and annotated regions. The results show that the SVGbit outperforms SPARK, SpatialDE and SOMDE in ranking individual SVGs (Fig. 2B). Generally, the SVGbit shows higher ARI values, especially at the top 1000 SVGs. In addition, the ARI values from SVGbit decrease along with ranking order, consistent with the expected ranking effect. By contrast, the ARI values from other methods exhibit great fluctuations along with ranking order in mouse brain (Fig. 2B), indicating the weak ability to rank the SVG aggregation pattern in this high-resolution dataset. The SPARK was excluded from comparison in this large dataset due to its slow speed. It should be noted that the selection of different ranking step does not impact the evaluation and comparison results (Supplementary Fig. 3). Taken together, SVGbit can capture the SVG aggregation patterns from spatial transcriptomics data at various resolutions.

### The individual SVG analysis in developing mouse brain

**Fig. 3:**
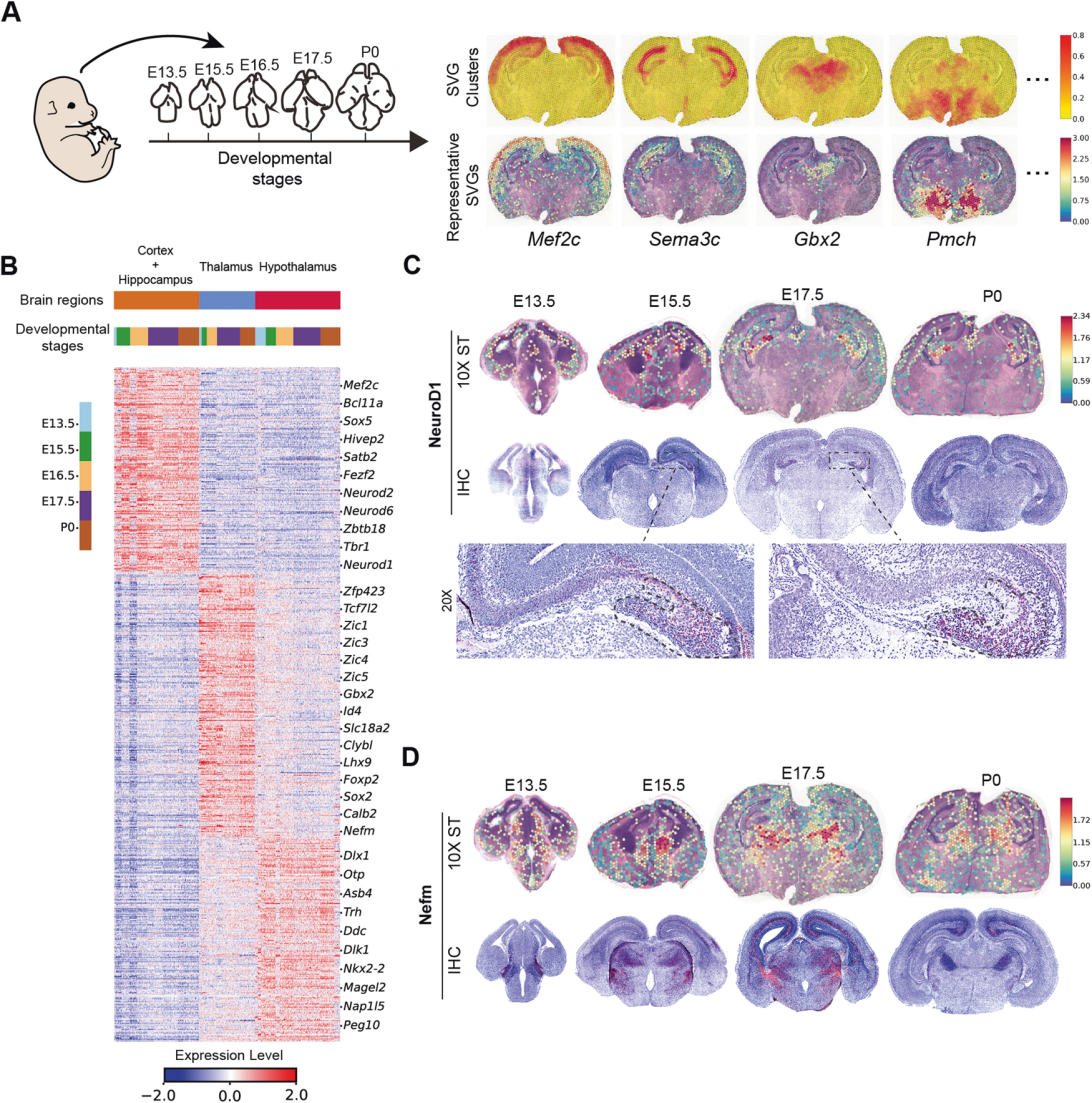
The spatiotemporal patterns of individual SVGs in mouse brain development. **A** The data generation and representative SVGs. The E17.5 replicate 1 was used as example for SVG clusters and representative genes. **B** The SVG expressions in the anatomic regions during developmental stages. The first color bar in the top denotes the three representative brain regions, while the second color bar in the top and the left color bar together indicate the developmental stages in each brain region. The pixel in each row and column represents the gene expression as shown in the bottom color bar. **C** The spatiotemporal dynamics of NeuroD1. **D** The spatiotemporal dynamics of Nefm.

We then investigated the developing mouse brain by generating spatial transcriptomics and IHC datasets for mouse brains at embryonic days 13.5, 15.5, 16.5, and 17.5 and postnatal day 0, which were denoted as E13.5, E15.5, E16.5, E17.5, and P0, respectively, in following statements (Fig. 3). Two replicates were generated in each time point, with pretty high reproducibility in aggregation index (Supplementary Figs. 4 and 5). In the spatial transcriptomics experiments, the frozen brain sections suffer from distortions, especially in the early developmental time points. This factor explains why the E13.5 show relatively lower reproducibility, and the technical replicates at E17.5 and P0 show relatively higher reproducibility.

We first focused on the top 500 SVGs in each replicate since this number of genes can capture the major region characteristics according to the previous analysis (Fig. 2). However, other SVG numbers can also be used. As shown in Fig. 3A, the selected SVGs and corresponding clusters coincide with well-known brain regions, such as neocortex, hippocampus, thalamus, and hypothalamus marked by the *Mef2c* expression [21], *Sema3c* expression [22], *Gbx2* expression [23], and *Pmch* expression [24], respectively. We also merged these top SVGs from 5 replicates (788 genes in total) and obtained many well-known region-specific marker genes (Fig. 3B). To validate the spatiotemporal dynamics of individual SVGs, we performed IHC staining on key proteins. In both spatial transcriptomics and IHC, the important factor NeuroD1 is mainly expressed around neocortex and ventricle at the early E13.5, and the expression is gradually located on hippocampus but with some expression in neocortex at later stages E15.5, E17.5, and P0 (Fig. 3C). This developmental pattern implies that NeuroD1 may play multiple roles in the mouse brain development, which is consistent with other works [25, 26]. As for the gene *Nefm*, its gene expression is mainly located on thalamus and hypothalamus at the early stage, but the expression in neocortex can also be observed during the development. The IHC results show the similar expression patterns though minor differences can be observed (Fig. 3D). The validations on other key SVGs are presented in following analyses.

### The combinatorial SVGs in developing mouse brain

**Fig. 4:**
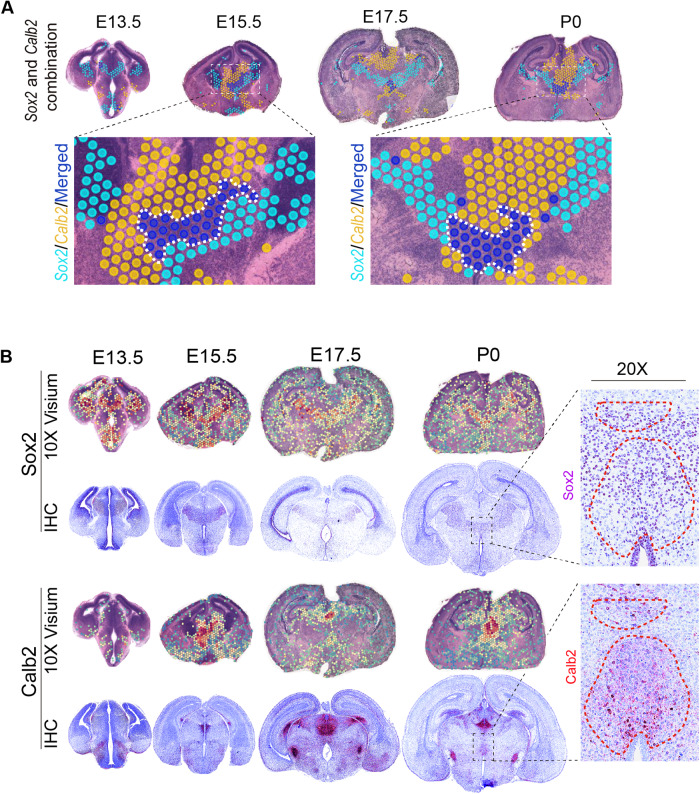
The Sox2 and Calb2 marking the spatiotemporal dynamics of three sub-regions in thalamic development. **A** The joint hotspot analysis between the two genes *Sox2* and *Calb2*. **B** The spatial gene expressions and IHC staining for Sox2 and Calb2, respectively.

We then investigated the combinatorial SVGs in the thalamus due to its importance in neuronal system as well as the absence of spatial-transcriptomics analysis in this region. Interestingly, the combinatorial ranking scores reveal that the pluripotent gene *Sox2* and developmental gene *Calb2* are largely exclusive, but gradually share the co-localized sub-region during the development. The joint hotspot analysis shows that these two genes separate the thalamic regions into three sub-regions since E15.5, including the *Sox2*-specific, *Calb2*-specific, and overlapped sub-regions (Fig. 4A). The gene *Sox2* is expressed in several regions at the early E13.5, including thalamus, hypothalamus, and ventricle, and then it is highly expressed like the V shape surrounding the thalamus in the later developmental points. As for the gene *Calb2*, it is not expressed in thalamus at early E13.5, but its expression can be detected at later stages (Fig. 4B). We validated this spatial relationship in protein level (Fig. 4B). Calretinin (*Calb2*) is one type of calcium-binding proteins which was initially isolated from the retina [27], and recent works have shown that this protein may play important roles in neural development [28]. One recent work revealed that the Slc18a2 expression is reduced when conditionally knocking out Sox2 in thalamus [29], which is consistent with the spatial co-localization among these genes (Supplementary Fig. 6). Thus, the sub-regional dynamics defined by genes *Sox2*, *Calb2*, and other genes may play important roles in thalamic development.

**Fig. 5:**
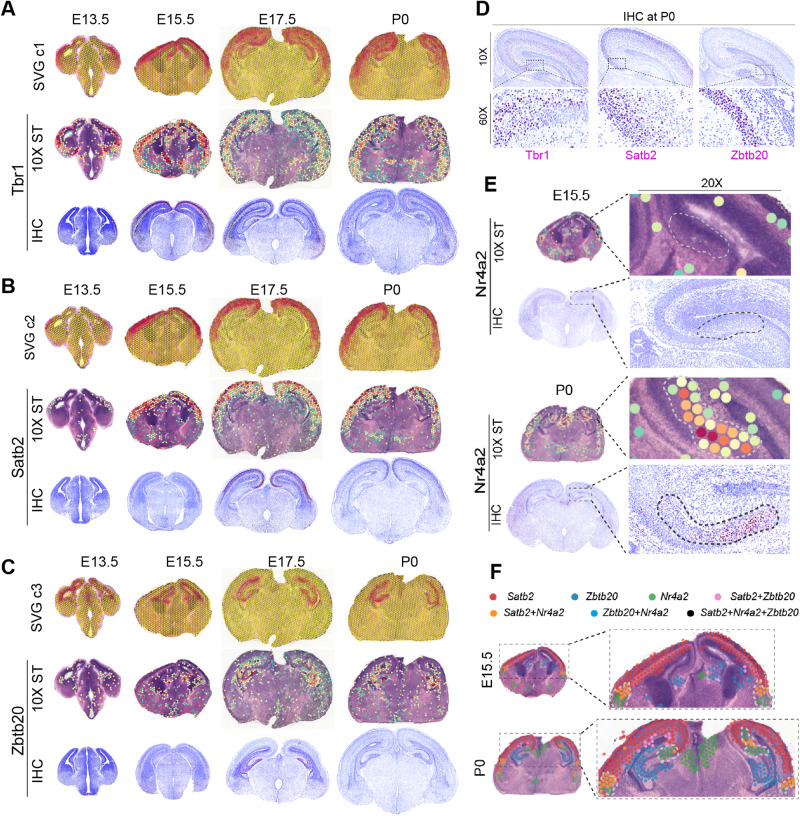
The combinatorial SVGs in neocortex and hippocampus. **A** SVGs expressed in both neocortex and hippocampus after hippocampus appearance. **B** SVGs mainly expressed in neocortex in the development. **C** SVGs mainly expressed in hippocampus in the development. **D** The higher-resolution IHC presentation for neocortex and hippocampus at P0. **E** The Nr4a2 expression patterns at E15.5 and P0. **F** The joint hotspot analysis among three genes *Satb2*, *Nr4a2*, and *Zbtb20*. In (**A**), (**B**), (**C**), and (**E**), the color schemes in SVG clusters and spatial transcriptomics are similar to that in Fig. 3A. In (**F**), the hotspot colors for different gene combinations are shown in the top legend.

We next applied SVGbit to neocortex and hippocampus regions. Generally, the neocortex and hippocampus SVGs can be grouped into 3 clusters (Fig. 5, Supplementary Table 5). One group of SVGs (SVG c1) are expressed in both neocortex and hippocampus after the hippocampus appearance, including the genes *Tbr1* and *Zbtb18* (Fig. 5A, Supplementary Fig. 7A). By contrast, another group of SVGs (SVG c2), including the *Satb2*, *Mef2c*, *Sox5*, *Sla*, and *9130024F11Rik* (Fig. 5B, Supplementary Fig. 7B), are mainly expressed in neocortex during the development, no matter whether the hippocampus appears or not. This pattern is mainly consistent with a recent immunostaining work showing that the cortical genes (*Mef2c* and *Sox5*) are not expressed in hippocampus [30]. In addition, the hippocampus SVGs (SVG c3) can also be detected, including the well-known gene *Zbtb20* (Fig. 5C, Supplementary Fig. 7C). In our data, the gene expressions in hippocampus are often observed in/around ventricle, partially due to the low spatial resolution and data variation in 10X Visium. We also performed IHC experiments on three representatives Tbr1, Satb2, and Zbtb20 to further validate these three developmental patterns in protein level (Fig. 5A–D). We then used SVGbit to calculate the combinatorial effects among the SVGs to explore the genes which may participate in separating the gene expression patterns between SVG c2 and SVG c3. As shown in Fig. 5E, we found that the gene *Nr4a2* is specifically expressed around the subiculum region between neocortex and hippocampus at P0 but not E15.5, and the IHC results confirm this observation in protein level. The joint hotspot analysis of the three genes *Satb2*, *Nr4a2,* and *Zbtb20* further shows that the *Nr4a2* expression can segregate the two representative genes for SVG c2 and c3 at P0 (Fig. 5F). Taken together, these results indicate that the Nr4a2^+^ neurons may be involved in segregating the neocortex and hippocampus during the development. However, further works are needed to explore regulatory mechanisms underlying these different developmental patterns.

## Discussion

Brain functions highly rely on the organization of spatial regions and corresponding marker genes, so it is important to detect the region-specific SVGs for brain regions. In this work, we developed a novel computational pipeline SVGbit to rank the individual and combinatorial SVGs to facilitate marker selection from spatial transcriptomics data. Our results also show that even part of top SVGs can capture the main characteristics of spatial domains and corresponding marker genes in various brain regions. When the ranking score decreases, the SVGs gradually lose the ability to distinguish brain regions. However, it should be noted that higher SVG ranking generally represents higher ability to mark anatomic regions and sub-regions, but higher ranking is not necessarily equal to more important functions.

By applying the SVGbit to our generated spatial transcriptomics and IHC data, this work reveals that the Sox2 and Calb2 can be used to define the spatiotemporal dynamics of three thalamic sub-regions, while the Nr4a2 has the ability to gradually mark the dynamical sub-region between neocortex and hippocampus during mouse brain development. These results suggest that SVGbit can be used to detect potentially novel marker genes from combinatorial SVGs. However, different gene combinations can be found to delineate the neuronal sub-populations. Further works are needed to validate these potential markers and investigate their functions.

Except for marking brain sub-regions and/or cell subtypes, the co-localized and exclusive SVGs may implicate biological relationships among these genes. In thalamus, the co-localized expression between genes *Sox2* and *Slc18a2* is consistent with their transcriptional regulation [29]. In neocortex and hippocampus, the genes *Satb2*, *Tbr1*, *Fezf2*, and *Bcl11b* show complicated expression relationship, including both co-localization and exclusion. This is also consistent with the genetic evidence that these genes exhibit complicated regulatory network [31]. Except for regulatory relationship, the spatial co-localization can also implicate the protein-protein interaction. For example, the co-localized FoxG1 and Zbtb18 form a protein complex to regulate neuronal migration and projection in neocortex [32]. These results suggest that the detected combinatorial SVGs can provide hints on their relationships, which may help further biological study.

In summary, we proposed a novel computational pipeline SVGbit to effectively rank the individual and combinatorial SVGs to facilitate key gene selection for brain regions, and we also used it to identify potentially novel marker genes and their combination sets for developing mouse brain. With the rapid development of spatial transcriptomics, this tool can be used or extended to other spatial transcriptomics data in the near future.

## Materials and methods

### Preparation for animal samples and 10X Visium libraries

The animal experiments were approved by the Institutional Animal Care and Use Committee, Yunnan University (YNUCARE20210001). Two-month-old C57BL/6J female mice (JAX, 000664) were mated to generate embryos and pups at embryonic day 13.5, 15.5, 16.5, 17.5, and postnatal day 0, which are denoted as E13.5, E15.5, E16.5, E17.5, and P0 in following analysis. The male brains were used for next experiments. Fresh brains were quickly dissected and frozen in liquid-nitrogen-cooled isopentane bath, embedded in OCT on dry ice, and then stored at −80 °C. The mouse brains at E13.5 were frozen by including the skulls since the tissues were quite soft in this time point. The mouse brains were cryosectioned at the thickness of 16 μm until the target regions were reached. Two biological replicates were included for E13.5, E15.5, and E16.5, and two technical replicates were included for E17.5 and P0 for the quality control, in which two continuous sections were discarded. The optimized permeation times for E13.5, E15.5, E16.5, E17.5, and P0 were 6, 8, 8, 8, and 8 min, respectively, and the original images were provided in supplementary Fig. 4. The RNA libraries were constructed by following 10X Visium protocol. Then the libraries were sequenced under Nova-seq PE150 platform.

### Data pre-processing

In the paired-end reads, the first 28 bp in the left-end reads containing the spatial barcodes and UMI were selected by using the software trimmomatic [33]. Then these 28 bp and right-end reads were used in the software Spaceranger (version 1.2.2) with default parameters by using mm10 as reference genome (Supplementary Table 1). The gene was removed from following analysis if its UMI was not detected in more than 1% spots. If the total UMIs in all spots were less than 10, the gene was also removed. The gene expressions in each spot were normalized by using the formula:

1 $$r_{ij}^\prime = \ln \left( {\frac{{r_{ij} \ast 10000}}{{\mathop {\sum}\nolimits_{j = 1}^n {r_{ij}} }} + 1} \right)$$

where *r*_*ij*_ represents the raw expression for gene *j* in spot *i*, and *n* is the gene number. The processed 10X Visium data for human dorsolateral prefrontal cortex was obtained from spatialLIBD [19], and the processed Stereo-seq dataset for mouse brain [18] was downloaded from CNGB with accession code CNP0001543.

### Ranking and clustering SVGs, and combination scores

For each gene, the local Moran’s I [34] was used to perform the hotspot analysis, i.e., the spot with significantly higher expressions compared to neighbor ones, with 0.05 as threshold by using the Benjamini–Hochberg procedure to calculate false discovery rate (FDR). Then the binary value was used to distinguish hotspots and non-hotspots by using following formula:

2 $$h_i = \left\{ {\begin{array}{*{20}{c}} {1,spot\,i \in hotspot\,set} \\ {0,spot\,i \,\notin \,hotspot\,set} \end{array}} \right.$$

For the given gene, the local aggregation density for selected hotspot *i* was defined by using following formula:

3 $$D_i = \mathop{\sum}\limits_{j = 1}^K {w_jh_j}$$

where *j* represents its K-nearest-neighbor (KNN, K = 6 in this work) spots, and *h*_*j*_ is the binary value calculated in formula (2). The weight *w*_*j*_ is defined as:

4 $$w_j = {\mathrm{log}}\left( {p_j} \right)/\mathop {\sum}\limits_{l = 1}^K \log \left( {p_l} \right)$$

where *p*_*l*_ is the *P*-value of spot *l* calculated from local Moran’s I and *l* denotes the KNN for the selected hotspot *i*. Then the aggregation index of each gene was calculated by averaging local aggregation densities of all hotspots:

5 $$AI_g = \mathop{\sum}\limits_{i = 1}^{n_g} {D_i/n_g}$$

where *n*_*g*_ is the hotspot number for gene *g*. It should be noted that the aggregation index is robust to the different KNN values in local aggregation density calculation (Supplementary Fig. 8).

The SVGs with the same spatial aggregation pattern can show great differences in gene expressions (Supplementary Fig. 9). To reduce the negative impact of this gene-expression variation, the aforementioned binary value *h*_*i*_ were used as input in SVG clustering, in which the hierarchical clustering was used with the Jaccard coefficient as the metric for SVG similarity. When visualizing the individual SVG cluster, the averaged hotspot frequency in each spot was calculated among all genes in the selected SVG cluster. In the thorough pattern analysis for the neocortex and hippocampus SVG clusters, we manually removed the genes which show obvious expressions in other regions to guarantee that the selected SVGs are mainly expressed in cortex and/or hippocampus. We also found that there were two subtypes in the neocortex SVGs and manually separated them. Due to the dynamical expression patterns and experimental variations, some SVGs can be occasionally clustered into different groups among samples, and then the major SVG clusters in all samples were used in heatmap presentation.

We mainly scored the combinatorial SVGs in the same or neighbor clusters to reduce searching space. Specifically, the co-localization and exclusion degrees between two SVGs were calculated by following scRNA-seq works [35, 36] with some modifications. For the selected SVGs and SVG clusters, let *X* denote the binary matrix with each entry calculated by using formula (2). Then the pair-wise co-localization among SVGs was calculated as:

6 $$C = X \cdot X^{\prime}$$

The pair-wise exclusion was calculated as:

7 $$E = (1-X) \cdot X^{\prime}$$

where 1 denotes the matrix with all entries as one. The co-localization and exclusion scores were used to rank the spatial co-localization and exclusion among SVGs.

### Immunohistochemistry

The dissected fresh brains were fixed in the 4% paraformaldehyde overnight at 4 °C, and then were dehydrated through ethanol gradients (50%, 75%, 85%, 95%, 100%) and xylenes from 30 min up to 2 h. Then brain tissues were embedded in paraffin after replacement of xylenes by paraffin. Sections were collected at the thickness of 5 μm and then were subjected to the immunostaining after deparaffinization. Antigen retrieval was carried out and sections were boiling in the 10 mM sodium-citrate solution (pH at 6.0) for 10 min. After that, sections were incubated in PBST (0.2% TritonX-100 in PBS) 1 h for the penetration and were further incubated in H_2_O_2_ for 10 min for bleaching non-specific HRP reaction. Animal-free blocking solution (CST, 15019) was used for the blocking, then primary antibodies (Supplementary Table 6) were mixed with 50 times diluted animal-free blocking solution, and further incubation was carried out at 4 °C overnight. After thoroughly washing, HRP-conjugated secondary antibodies were incubated in PBS at room temperature for 40 min. DAB staining substrates were incubated from 1 min up to 30 min, until clearly target protein shown up. DAB substances were incubated exact same time for one specific primary antibody across all stages. Images were captured by SQS-1000 high-resolution regular light scanning microscope at ×400 magnification.

## Supplementary information

Supplemental Materials

## Data Availability

The data in this work, including the raw sequencing reads and H&E images in spatial transcriptomics (10X Visium) and IHC images are deposited in National Genomics Data Center (https://ngdc.cncb.ac.cn) with the BioProject accession number PRJCA010498, in which the sequencing reads are under GSA accession number CRA007506, the H&E images are under OMIX accession number OMIX001298 and the IHC images are under OMIX accession number OMIX001319. The serial numbers and sample regions of the Visium slides in Supplementary Table 1 can be used to download slide layout files in 10X Genomics when running spaceranger. The software SVGbit is written in Python, and freely available at GitHub (https://github.com/CPenglab/svgbit) and PyPI (https://pypi.org/project/svgbit/).

## References

[1] La Manno G, Siletti K, Furlan A, Gyllborg D, Vinsland E, Mossi Albiach A (2021). Molecular architecture of the developing mouse brain. Nature..

[2] Rao A, Barkley D, Franca GS, Yanai I (2021). Exploring tissue architecture using spatial transcriptomics. Nature..

[3] Cheng M, Jiang Y, Xu J, Mentis AA, Wang S, Zheng H, et al. Spatially resolved transcriptomics: a comprehensive review of their technological advances, applications, and challenges. J Genet Genomics. 2023. 10.1016/j.jgg.2023.03.011. 10.1016/j.jgg.2023.03.011 36990426

[4] Ortiz C, Navarro JF, Jurek A, Martin A, Lundeberg J, Meletis K (2020). Molecular atlas of the adult mouse brain. Sci Adv.

[5] Di Bella DJ, Habibi E, Stickels RR, Scalia G, Brown J, Yadollahpour P (2021). Molecular logic of cellular diversification in the mouse cerebral cortex. Nature..

[6] Eng CL, Lawson M, Zhu Q, Dries R, Koulena N, Takei Y (2019). Transcriptome-scale super-resolved imaging in tissues by RNA seqFISH. Nature..

[7] Stickels RR, Murray E, Kumar P, Li J, Marshall JL, Di Bella DJ (2021). Highly sensitive spatial transcriptomics at near-cellular resolution with Slide-seqV2. Nat. Biotechnol.

[8] Fang R, Xia C, Close JL, Zhang M, He J, Huang Z (2022). Conservation and divergence of cortical cell organization in human and mouse revealed by MERFISH. Science..

[9] Edsgard D, Johnsson P, Sandberg R (2018). Identification of spatial expression trends in single-cell gene expression data. Nat Methods.

[10] Svensson V, Teichmann SA, Stegle O (2018). SpatialDE: identification of spatially variable genes. Nat Methods.

[11] Sun S, Zhu J, Zhou X (2020). Statistical analysis of spatial expression patterns for spatially resolved transcriptomic studies. Nat Methods.

[12] Hao M, Hua K, Zhang X (2021). SOMDE: a scalable method for identifying spatially variable genes with self-organizing map. Bioinformatics..

[13] Zhao E, Stone MR, Ren X, Guenthoer J, Smythe KS, Pulliam T (2021). Spatial transcriptomics at subspot resolution with BayesSpace. Nat Biotechnol.

[14] Hu J, Li X, Coleman K, Schroeder A, Ma N, Irwin DJ (2021). SpaGCN: integrating gene expression, spatial location and histology to identify spatial domains and spatially variable genes by graph convolutional network. Nat Methods.

[15] Yuan Z, Li Y, Shi M, Yang F, Gao J, Yao J (2022). SOTIP is a versatile method for microenvironment modeling with spatial omics data. Nat Commun.

[16] Shang L, Zhou X (2022). Spatially aware dimension reduction for spatial transcriptomics. Nat Commun.

[17] Dong K, Zhang S (2022). Deciphering spatial domains from spatially resolved transcriptomics with an adaptive graph attention auto-encoder. Nat Commun.

[18] Chen A, Liao S, Cheng M, Ma K, Wu L, Lai Y (2022). Spatiotemporal transcriptomic atlas of mouse organogenesis using DNA nanoball-patterned arrays. Cell..

[19] Maynard KR, Collado-Torres L, Weber LM, Uytingco C, Barry BK, Williams SR (2021). Transcriptome-scale spatial gene expression in the human dorsolateral prefrontal cortex. Nat Neurosci.

[20] Hubert L, Arabie P (1985). Comparing partitions. J Classif.

[21] Leifer D, Krainc D, Yu YT, Mcdermott J, Breitbart RE, Heng J (1993). MEF2C, a MADS MEF2-family transcription factor expressed in a laminar distribution in cerebral-cortex. Proc Natl Acad Sci USA.

[22] Steup A, Lohrum M, Hamscho N, Savaskan NE, Ninnemann O, Nitsch R (2000). Sema3C and Netrin-1 differentially affect axon growth in the hippocampal formation. Mol Cell Neurosci.

[23] Chen L, Guo QX, Li JYH (2009). Transcription factor Gbx2 acts cell-nonautonomously to regulate the formation of lineage-restriction boundaries of the thalamus. Development..

[24] Beekly BG, Frankel WC, Berg T, Allen SJ, Garcia-Galiano D, Vanini G (2020). Dissociated Pmch and Cre expression in lactating Pmch-Cre BAC transgenic mice. Front Neuroanat.

[25] Gao Z, Ure K, Ables JL, Lagace DC, Nave KA, Goebbels S (2009). Neurod1 is essential for the survival and maturation of adult-born neurons. Nat Neurosci.

[26] Singh A, Mahesh A, Noack F, Cardoso de Toledo B, Calegari F, Tiwari VK (2022). Tcf12 and NeuroD1 cooperatively drive neuronal migration during cortical development. Development..

[27] Rogers JH (1987). Calretinin—a gene for a novel calcium-binding protein expressed principally in neurons. J Cell Biol.

[28] Qi YB, Cheng HM, Wang Y, Chen Z (2022). Revealing the precise role of calretinin neurons in epilepsy: we are on the way. Neurosci Bull.

[29] Mercurio S, Serra L, Motta A, Gesuita L, Sanchez-Arrones L, Inverardi F (2019). Sox2 acts in thalamic neurons to control the development of retina-thalamus-cortex connectivity. iScience.

[30] Zhang L, Song NN, Zhang Q, Mei WY, He CH, Ma PC (2020). Satb2 is required for the regionalization of retrosplenial cortex. Cell Death Differ.

[31] Srinivasan K, Leone DP, Bateson RK, Dobreva G, Kohwi Y, Kohwi-Shigematsu T (2012). A network of genetic repression and derepression specifies projection fates in the developing neocortex. Proc Natl Acad Sci USA.

[32] Cargnin F, Kwon JS, Katzman S, Chen B, Lee JW, Lee SK (2018). FOXG1 orchestrates neocortical organization and cortico-cortical connections. Neuron..

[33] Bolger AM, Lohse M, Usadel B (2014). Trimmomatic: a flexible trimmer for Illumina sequence data. Bioinformatics..

[34] Anselin L (1995). Local indicators of spatial association—LISA. Geogr Anal.

[35] Delaney C, Schnell A, Cammarata LV, Yao-Smith A, Regev A, Kuchroo VK (2019). Combinatorial prediction of marker panels from single-cell transcriptomic data. Mol Syst Biol.

[36] Wang F, Liang S, Kumar T, Navin N, Chen K (2019). SCMarker: ab initio marker selection for single cell transcriptome profiling. PLoS Comput Biol.

