# Supplementary material for: The spatiotemporal dynamics of spatially variable genes in developing mouse brain revealed by a novel computational scheme
Source: Cell Death Discov. 2023 Jul 27;9:264. doi: 10.1038/s41420-023-01569-w (PMC10374563; doi:10.1038/s41420-023-01569-w)
Supplement: Supplementary file 1 — Supplemental Materials [file 41420_2023_1569_MOESM1_ESM.pdf]

Supplementary Materials

The spatiotemporal dynamics of spatially variable genes in developing mouse brain revealed by a novel computational scheme

Yingzhou Hong[#], Kai Song[#], Zongbo Zhang[#], Yuxia Deng, Xue Zhang, Jinqian Zhao, Jun Jiang, Qing Zhang, Chunming Guo[*], Cheng Peng[*]

Center for Life Sciences, School of Life Sciences, Yunnan University, Kunming, 650500, China

#Co-first authors.

*To whom correspondence should be addressed:

Cheng Peng, Email: chengpeng@ynu.edu.cn

Chunming Guo, Email: chunmingguo@ynu.edu.cn

Key words: spatially variable gene, spatial transcriptomics, marker, brain

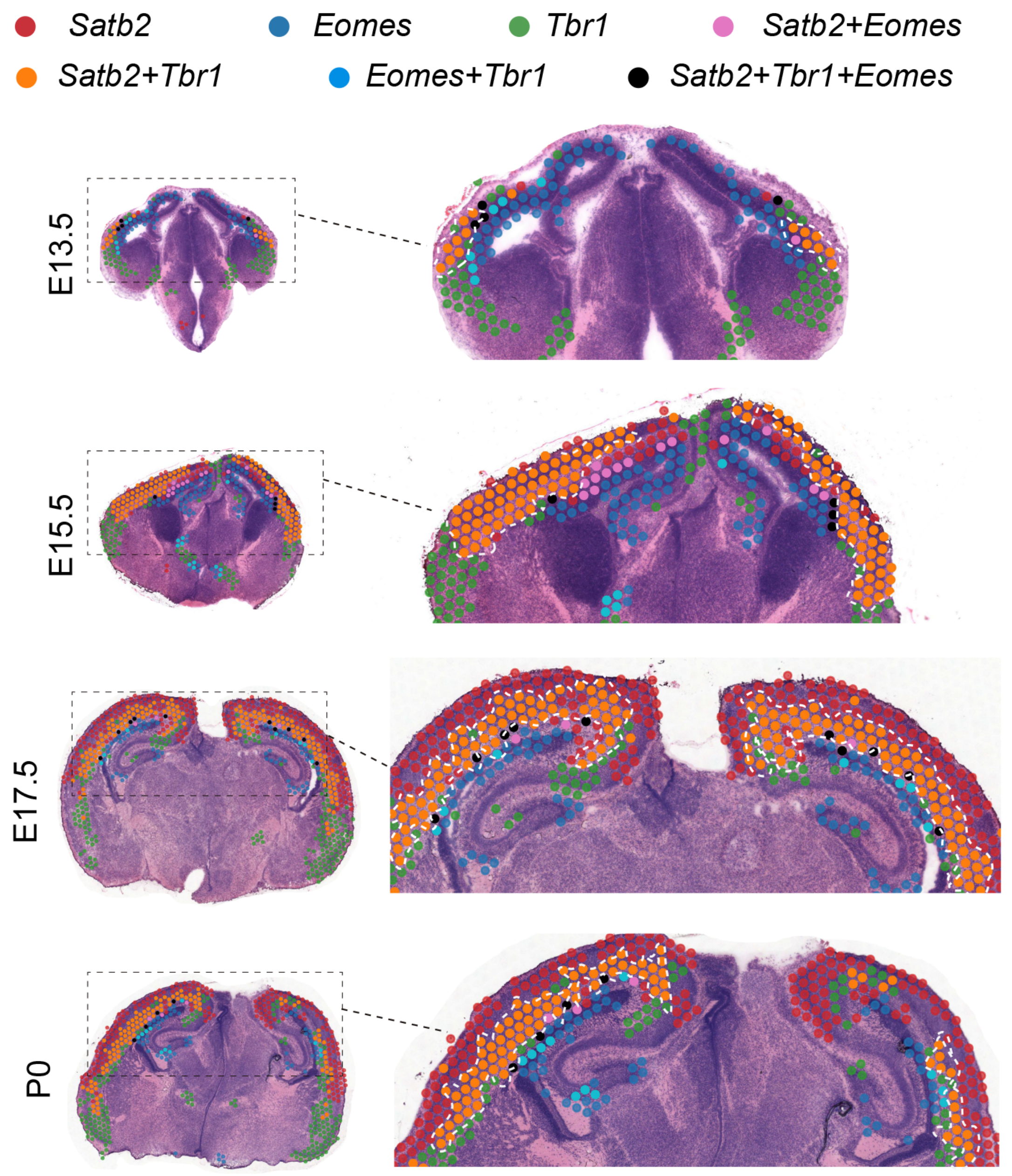

Supplementary figure 1. The combinations among genes *Satb2*, *Tbr1* and *Eomes* during mouse brain development. The data were generated in this work.

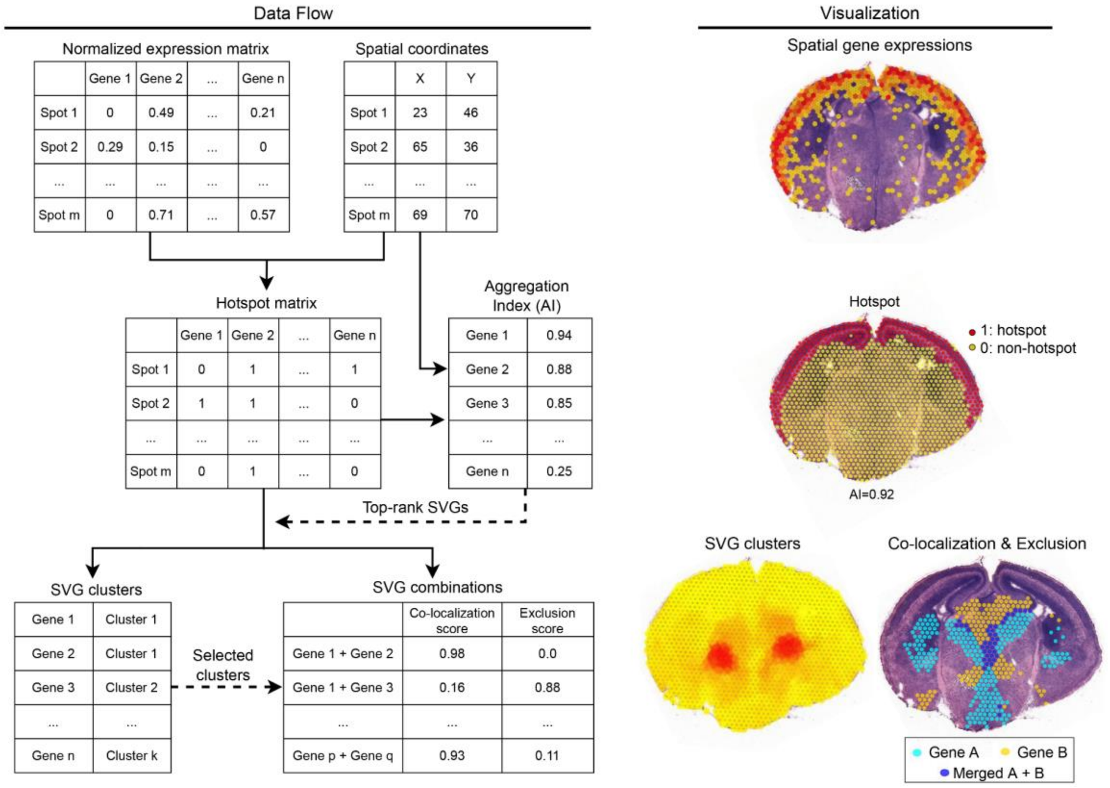

Supplementary figure 2. The main data flow in SVGbit. In the left subfigures, the solid arrows indicate the data flow from input to output, and the dashed arrows indicate that only part of the selected SVGs or SVG clusters are used as input. The right subfigures show the visualizations corresponding to those in Figure 1.

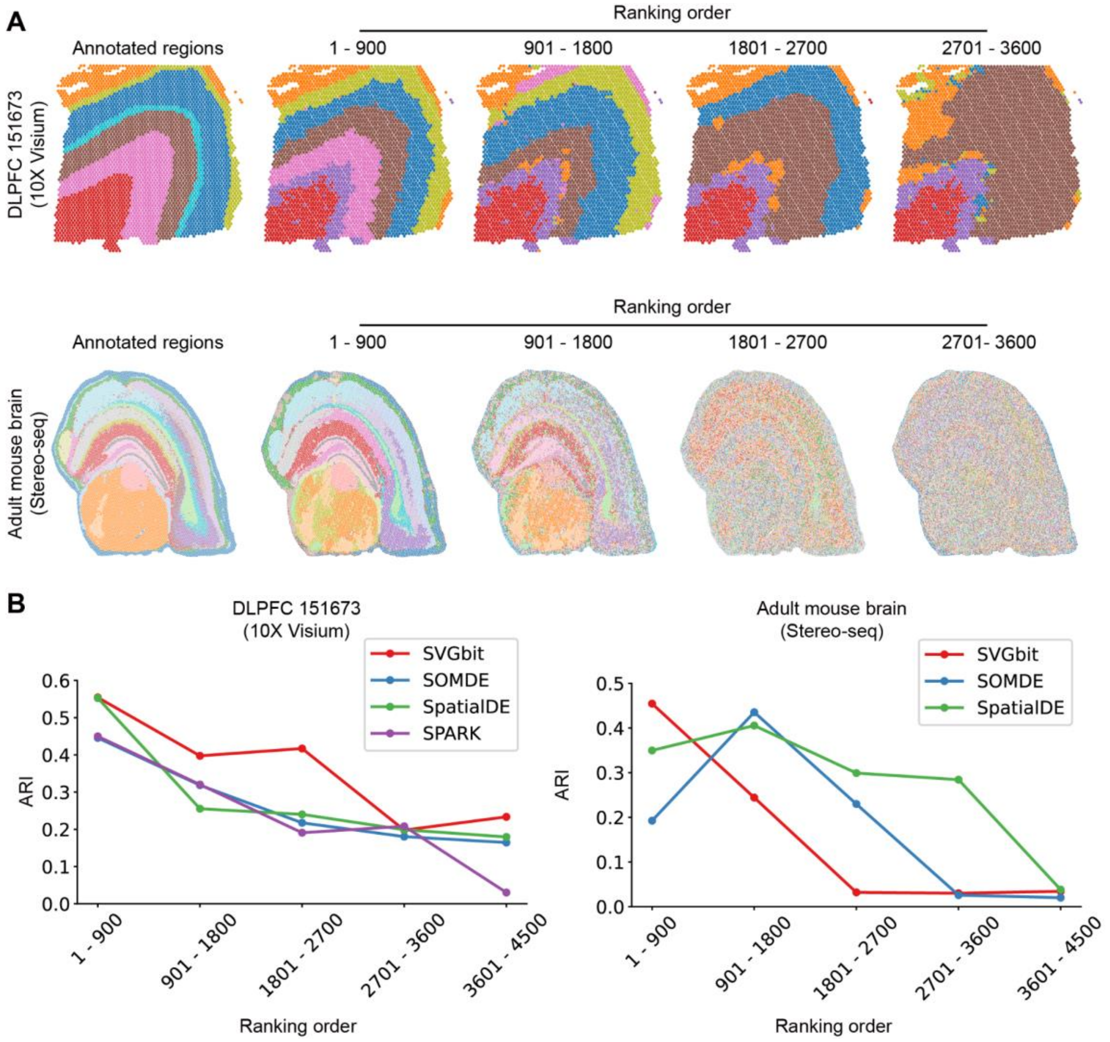

Supplementary figure 3. Evaluation on the SVGbit with step 800. (A) The spatial domains along with ranking order. (B) The comparisons among different SVG detection methods.

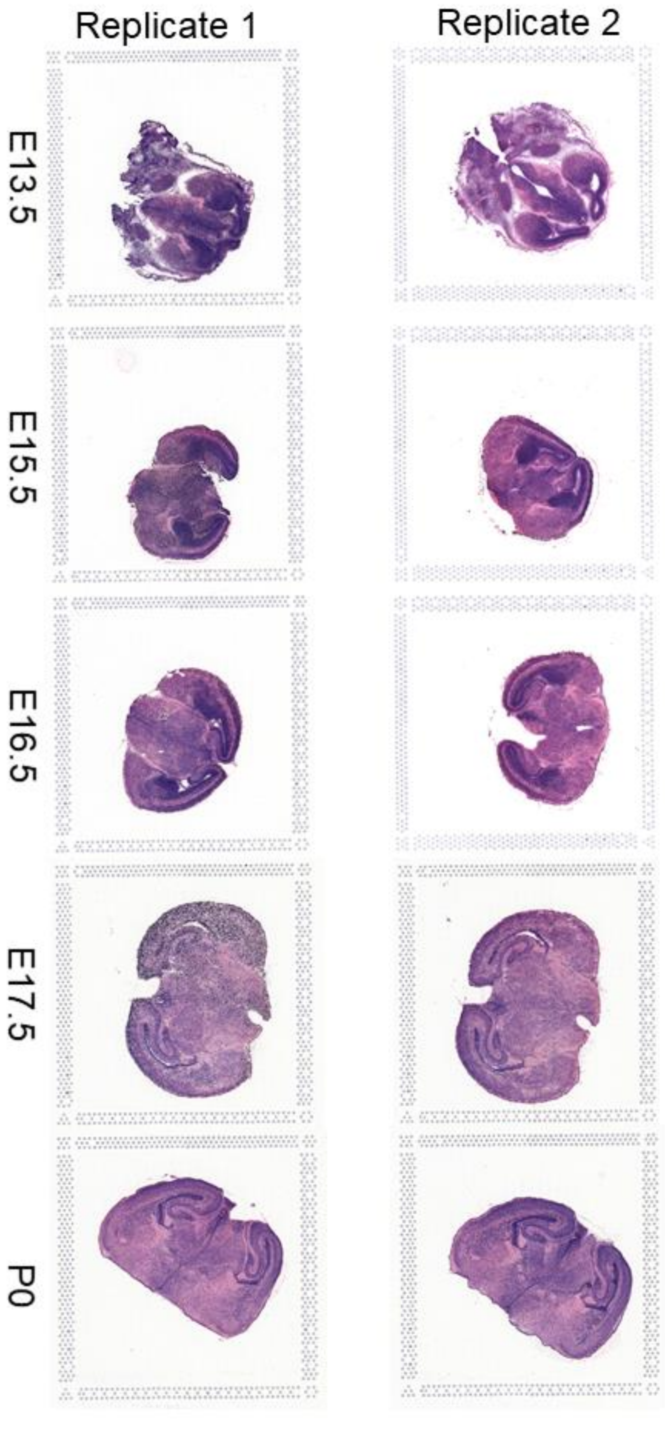

Supplementary figure 4. The H&E images for 10 replicates in our dataset. These images are used as background for spatial expression, SVG cluster and spatial domain presentations, which are rearranged for better presentation. The simplified statement on E13.5, E15.5, E16.5, E17.5 and P0 represents E13.5 replicate 2, E15.5 replicate 2, E16.5 replicate 2, E17.5 replicate 1 and P0 replicate 1 in this manuscript.

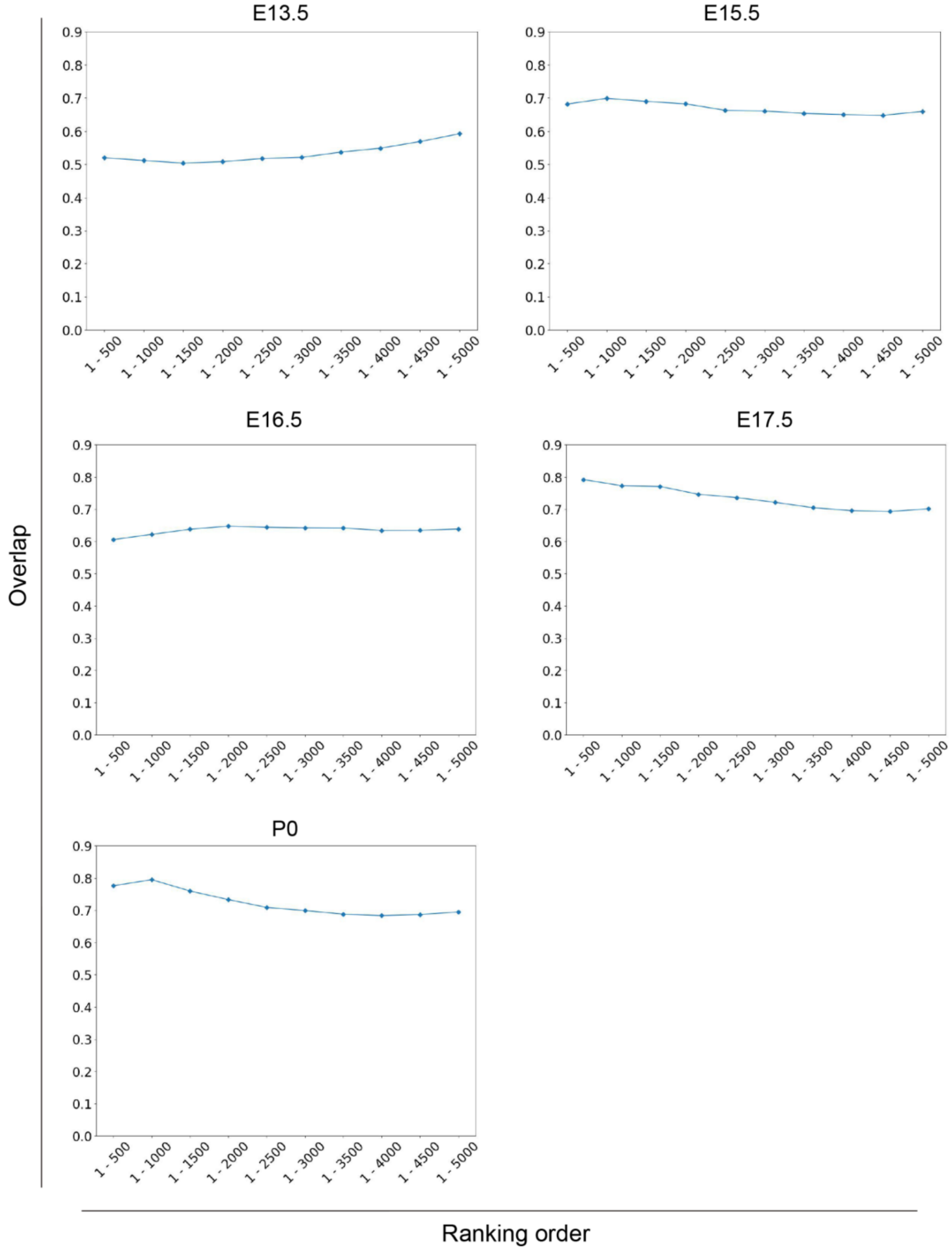

Supplementary figure 5. The reproducibility of SVG ranking scheme in E13.5, E15.5, E16.5, E17.5 and P0. The x axis denotes the ranking order of selected SVGs, and the y axis denotes the overlap ratio calculated by the number of overlapped SVGs between replicates dividing the number of selected SVGs.

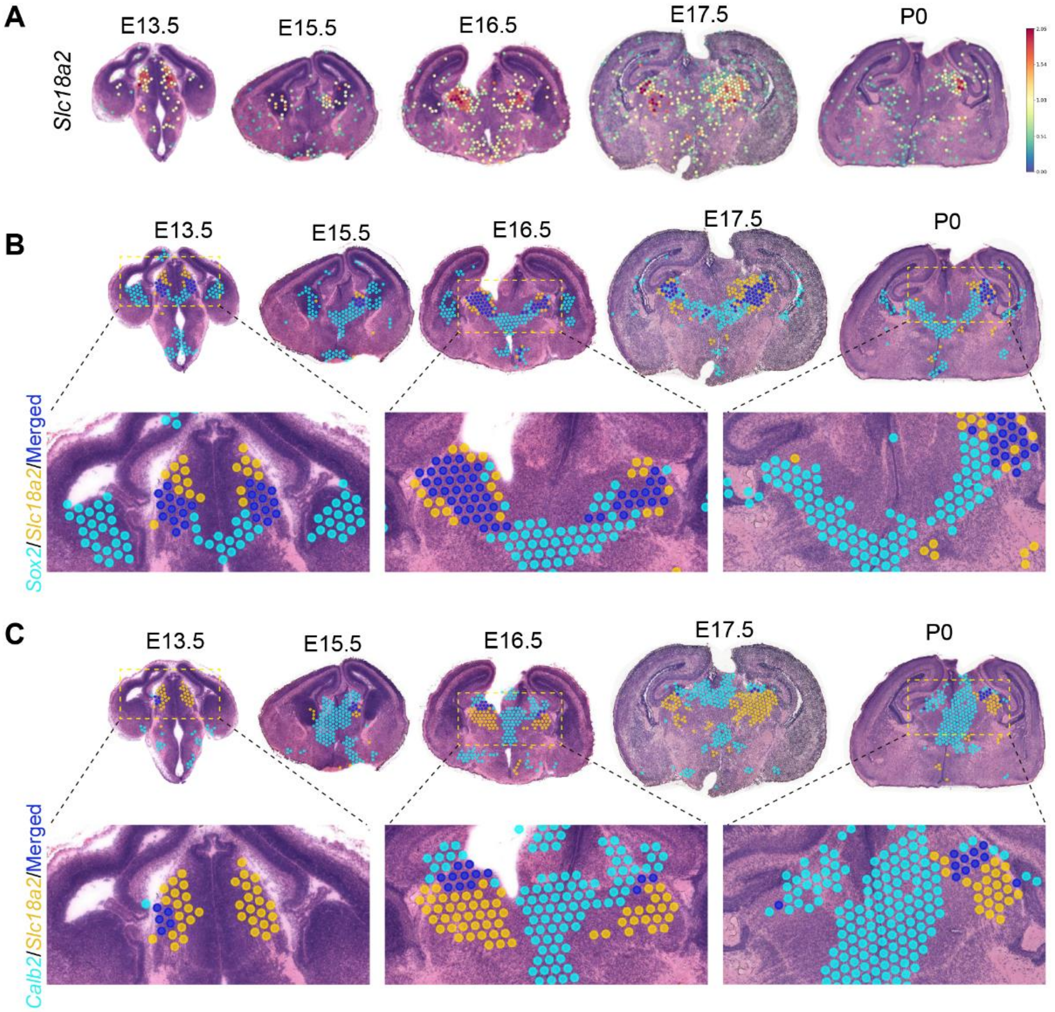

Supplementary figure 6. The relationship among genes *Sox2*, *Calb2* and *Slc18a2* in thalamus. (A) The spatial expressions of gene *Slc18a2* in different time points. (B) The joint hotspot analysis between *Sox2* and *Slc18a2*. (C) The joint hotspot analysis between *Calb2* and *Slc18a2*.

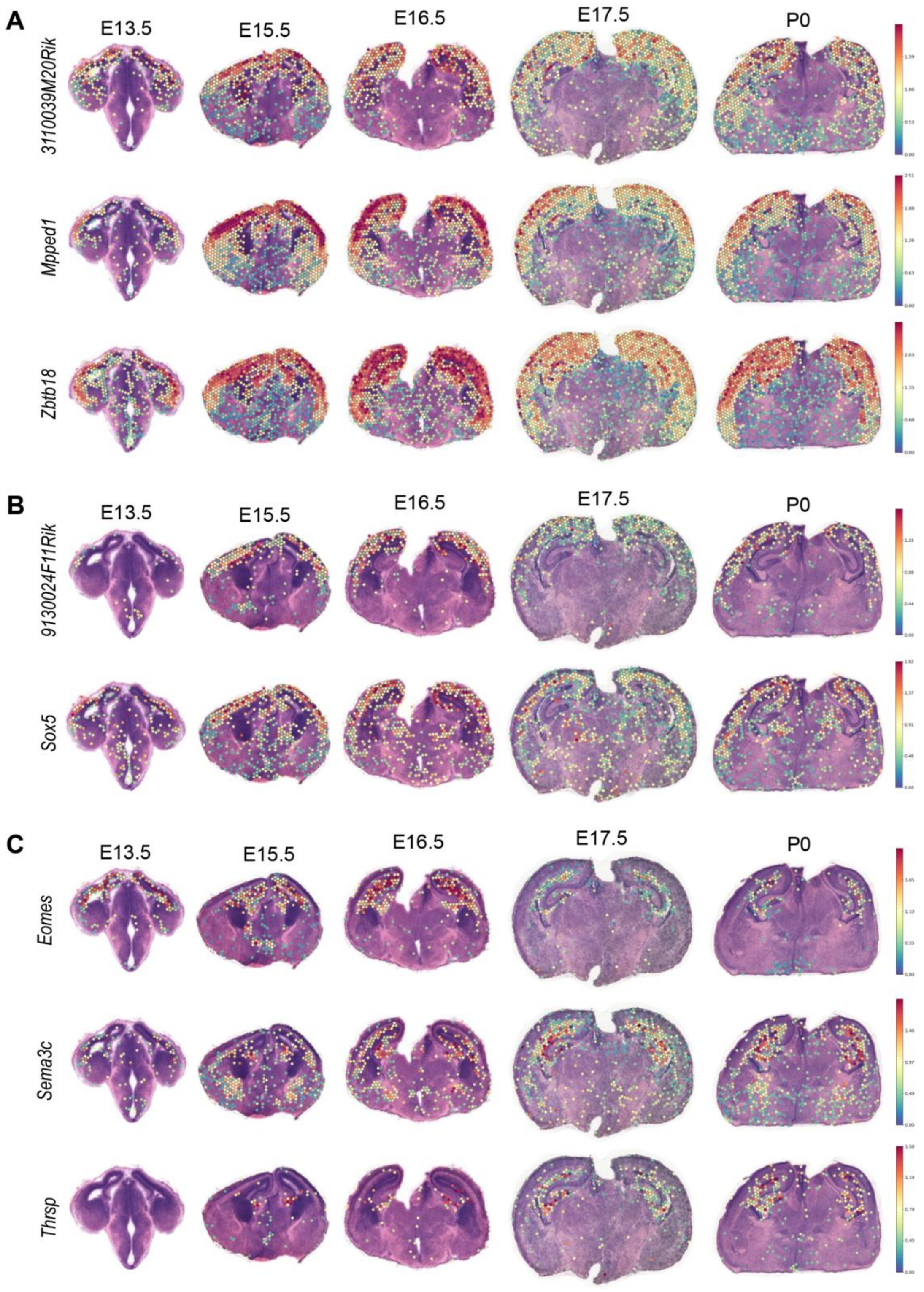

Supplementary figure 7. Spatial gene expressions of neocortex and hippocampus SVGs. (A), (B) and (C) show the examples for SVG c1, c2 and c3 respectively.

E13.5

Replicate 1

K	4	5	6	7	8	9	10	11	12
12	0.88	0.92	0.95	0.96	0.97	0.98	0.98	0.99	1
11	0.89	0.92	0.95	0.96	0.97	0.98	0.99	1	0.99
10	0.89	0.93	0.96	0.97	0.98	0.99	1	0.99	0.98
9	0.9	0.93	0.96	0.97	0.99	1	0.99	0.98	0.98
8	0.91	0.94	0.96	0.98	1	0.99	0.98	0.97	0.97
7	0.91	0.95	0.97	1	0.98	0.97	0.97	0.96	0.96
6	0.92	0.96	1	0.97	0.96	0.96	0.96	0.95	0.95
5	0.94	1	0.96	0.95	0.94	0.93	0.93	0.92	0.92
4	1	0.94	0.92	0.91	0.91	0.9	0.89	0.89	0.88

Replicate 2

K	4	5	6	7	8	9	10	11	12
12	0.89	0.92	0.95	0.96	0.97	0.98	0.99	0.99	1
11	0.9	0.93	0.95	0.97	0.98	0.98	0.99	1	0.99
10	0.9	0.93	0.96	0.97	0.98	0.99	1	0.99	0.99
9	0.9	0.94	0.96	0.98	0.99	1	0.99	0.98	0.98
8	0.91	0.94	0.97	0.98	1	0.99	0.98	0.98	0.97
7	0.92	0.95	0.98	1	0.98	0.98	0.97	0.97	0.96
6	0.93	0.96	1	0.98	0.97	0.96	0.96	0.95	0.95
5	0.95	1	0.96	0.95	0.94	0.94	0.93	0.93	0.92
4	1	0.95	0.93	0.92	0.91	0.9	0.9	0.9	0.89

E15.5

Replicate 1

K	4	5	6	7	8	9	10	11	12
12	0.88	0.91	0.94	0.96	0.97	0.98	0.98	0.99	1
11	0.88	0.92	0.95	0.96	0.97	0.98	0.99	1	0.99
10	0.89	0.92	0.95	0.97	0.98	0.99	1	0.99	0.98
9	0.89	0.93	0.96	0.97	0.98	1	0.99	0.98	0.98
8	0.9	0.94	0.96	0.98	1	0.98	0.98	0.97	0.97
7	0.91	0.94	0.97	1	0.98	0.97	0.97	0.96	0.96
6	0.92	0.96	1	0.97	0.96	0.96	0.95	0.95	0.94
5	0.94	1	0.96	0.94	0.94	0.93	0.92	0.92	0.91
4	1	0.94	0.92	0.91	0.9	0.89	0.89	0.88	0.88

Replicate 2

K	4	5	6	7	8	9	10	11	12
12	0.84	0.89	0.93	0.94	0.96	0.96	0.97	0.99	1
11	0.85	0.9	0.93	0.95	0.96	0.97	0.98	1	0.99
10	0.85	0.9	0.94	0.95	0.97	0.98	1	0.98	0.97
9	0.86	0.91	0.94	0.96	0.98	1	0.98	0.97	0.96
8	0.87	0.92	0.95	0.97	1	0.98	0.97	0.96	0.96
7	0.88	0.93	0.96	1	0.97	0.96	0.95	0.95	0.94
6	0.89	0.94	1	0.96	0.95	0.94	0.94	0.93	0.93
5	0.92	1	0.94	0.93	0.92	0.91	0.9	0.9	0.89
4	1	0.92	0.89	0.88	0.87	0.86	0.85	0.85	0.84

E16.5

Replicate 1

K	4	5	6	7	8	9	10	11	12
12	0.9	0.93	0.96	0.97	0.97	0.98	0.99	0.99	1
11	0.91	0.93	0.96	0.97	0.98	0.98	0.99	1	0.99
10	0.91	0.94	0.96	0.97	0.98	0.99	1	0.99	0.99
9	0.91	0.94	0.97	0.98	0.99	1	0.99	0.98	0.98
8	0.92	0.95	0.97	0.98	1	0.99	0.98	0.98	0.97
7	0.93	0.95	0.98	1	0.98	0.98	0.97	0.97	0.97
6	0.93	0.96	1	0.98	0.97	0.97	0.96	0.96	0.96
5	0.95	1	0.96	0.95	0.95	0.94	0.94	0.93	0.93
4	1	0.95	0.93	0.93	0.92	0.91	0.91	0.91	0.9

Replicate 2

K	4	5	6	7	8	9	10	11	12
12	0.84	0.89	0.93	0.94	0.96	0.97	0.98	0.99	1
11	0.85	0.9	0.93	0.95	0.96	0.97	0.98	1	0.99
10	0.85	0.9	0.94	0.95	0.97	0.98	1	0.98	0.98
9	0.86	0.91	0.94	0.96	0.98	1	0.98	0.97	0.97
8	0.87	0.92	0.95	0.97	1	0.98	0.97	0.96	0.96
7	0.88	0.93	0.96	1	0.97	0.96	0.95	0.95	0.94
6	0.89	0.94	1	0.96	0.95	0.94	0.94	0.93	0.93
5	0.92	1	0.94	0.93	0.92	0.91	0.9	0.9	0.89
4	1	0.92	0.89	0.88	0.87	0.86	0.85	0.85	0.84

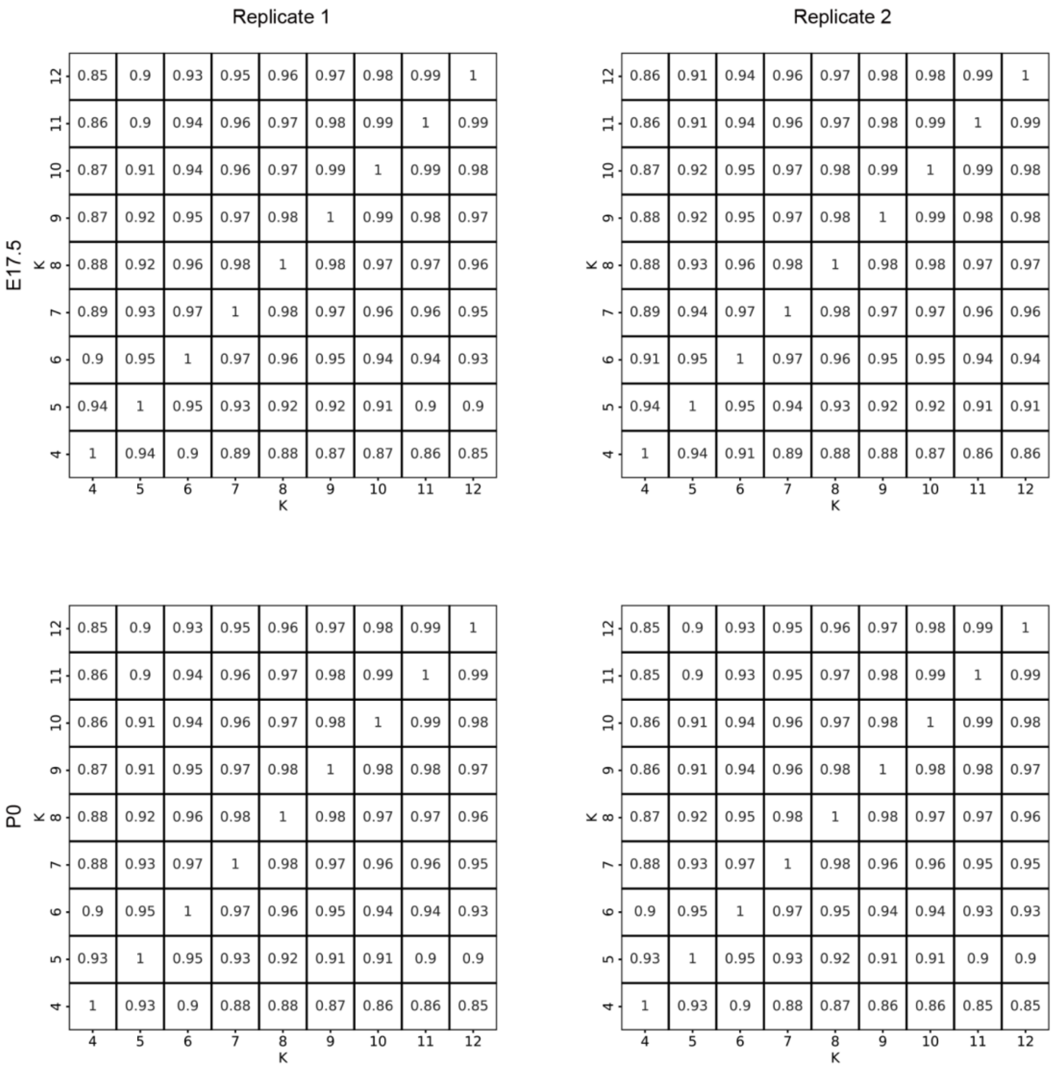

Supplementary figure 8. The Pearson correlation coefficients among aggregation indexes calculated by using different K nearest neighbors.

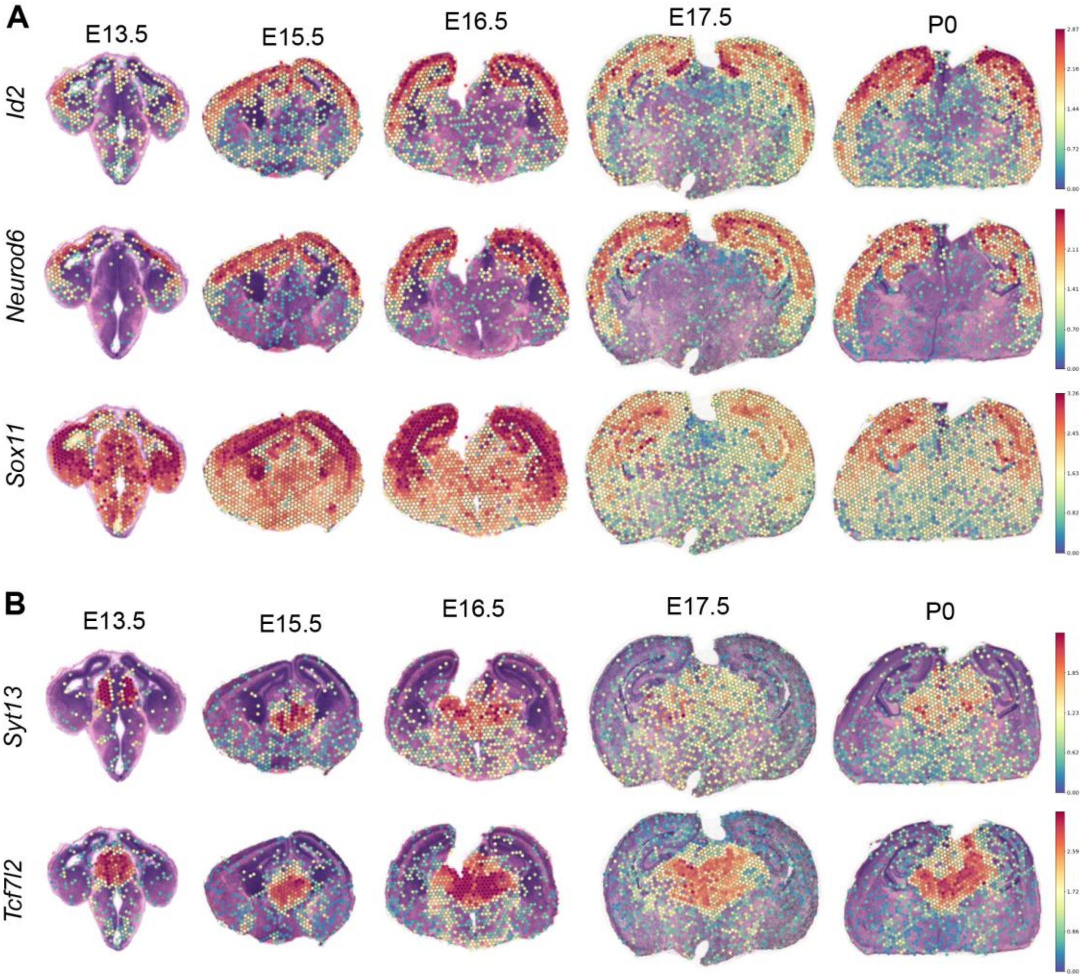

Supplementary figure 9. The spatial gene expressions showing similar aggregation patterns but with obvious variations. (A) Spatial gene expressions in neocortex and/or hippocampus. (B) Spatial gene expressions in thalamus.

Supplementary table 1. The data summary reported by Spaceranger. The spatial transcriptomics data were generated by using 10X Visium, and the serial numbers and regions in the Visium Spatial Gene Expression Slides are also given in the table.

**Sample Name**	**Short name in manuscript**	**Spots under tissue**	**Total Genes**	**Total Reads**	**Total UMIs**	**Valid Barcodes**	**Valid UMIs**	**Serial number**	**Region**
E13.5_rep1	-	1,563	20,151	283,533,191	22,393,070	97.1%	99.9%	V10M17-100	A1
E13.5_rep2	E13.5	1,694	19,471	120,859,907	21,234,159	97.8%	100%	V10M17-085	A1
E15.5_rep1	-	1,168	19,557	260,963,466	30,227,474	97.5%	100%	V10M17-100	B1
E15.5_rep2	E15.5	1,029	18,757	113,954,091	17,155,922	97.9%	100%	V10M17-085	B1
E16.5_rep1	-	1,355	20,430	276,649,234	46,590,981	97.4%	100%	V10M17-100	C1
E16.5_rep2	E16.5	1,314	18,532	115,496,997	13,503,579	97.8%	100%	V10M17-085	C1
E17.5_rep1	E17.5	2,205	20,136	199,094,255	29,355,267	97.9%	100%	V10M17-101	A1
E17.5_rep2	-	2,165	20,038	313,380,331	30,783,829	97.8%	100%	V10M17-101	B1
P0_rep1	P0	1,854	20,172	246,429,084	27,428,030	97.9%	100%	V10M17-101	C1
P0_rep2	-	1,905	19,906	210,597,285	24,134,416	97.9%	100%	V10M17-101	D1

Supplementary table 2. The summary of SVG detections from SPARK, SpatialDE and SOMDE. The second, third, fourth and fifth columns represent the reported minimum adjusted P value or Q value, the gene number with the minimum P/Q value, and the gene number with adjusted P value or Q value less than or equal to 0.05. No SVGs are detected in SOMDE for E13.5 two replicates. The softwares were run by using the parameters given in the online documents with the versions in supplementary table 3.

	Sample	Minimum adjusted P value	Gene number (Adjusted P value= minimum value)	Gene number (Adjusted P value <= 0.05)
SPARK	E135_Rep1	$1.01*10^{-14}$	487	2,266
	E135_Rep2	$8.88*10^{-15}$	554	2,958
	E155_Rep1	$2.72*10^{-15}$	2,280	6,692
	E155_Rep2	$4.33*10^{-15}$	1,310	4,799
	E165_Rep1	$1.96*10^{-15}$	3,333	8,483
	E165_Rep2	$4.22*10^{-15}$	1,189	4,239
	E175_Rep1	$2.84*10^{-15}$	1,698	5,257
	E175_Rep2	$2.69*10^{-15}$	1,883	5,648
	P0_Rep1	$3.14*10^{-15}$	1,601	5,254
	P0_Rep2	$3.10*10^{-15}$	1,508	4,627
	Sample	Minimum Q value	Gene number (Q value=minimum value)	Gene number (Q value <= 0.05)
SpatialDE	E135_Rep1	0	195	1,363
	E135_Rep2	0	397	2,808
	E155_Rep1	0	1,570	7,905
	E155_Rep2	0	734	4,166
	E165_Rep1	0	2,022	8,810
	E165_Rep2	0	897	4,518
	E175_Rep1	0	1,073	5,485
	E175_Rep2	0	1,362	5,908
	P0_Rep1	0	1,144	4,713
	P0_Rep2	0	1,112	4,385
	Sample	Minimum Q value	Gene number (Q value= minimum value)	Gene number (Q value <= 0.05)
SOMDE	E135_Rep1	0.428	3	0
	E135_Rep2	0.581	1	0
	E155_Rep1	$3.67*10^{-6}$	1	567
	E155_Rep2	0.0001	1	146
	E165_Rep1	$4.18*10^{-9}$	2	2,050
	E165_Rep2	$5.50*10^{-8}$	1	615
	E175_Rep1	$1.57*10^{-12}$	1	666
	E175_Rep2	0	1	1,290
	P0_Rep1	$9.28*10^{-10}$	1	791
	P0_Rep2	$3.38*10^{-10}$	1	600

Supplementary table 3. The software versions and links.

Software	Version	Link
SPARK	1.1.1	https://xzhoulab.github.io/SPARK/
SpatialDE	1.1.3	https://pypi.org/project/SpatialDE/
SOMDE	0.1.8	https://pypi.org/project/somde/
SVGbit	0.2.11	https://pypi.org/project/svgbit/<br>https://github.com/CPenglab/svgbit

Supplementary table 4. The summary of running times. Five cores were used in SPARK and SVGbit since these two softwares support parallel computing. In the online documents, there are no statements on the parallel-computing parameters when running the softwares SpatialDE and SOMDE. The SPARK was excluded from running-time statistics in the Stereo-seq data due to its slow speed in this large dataset. CPU: Intel Xeon Gold 6248. h: hour; m: minute; s: second.

	SPARK	SpatialDE	SOMDE	SVGbit
E135_Rep1	19m:34s	8m:57s	8m:27s	10m:26s
E135_Rep2	18m:23s	10m:1s	8m:5s	11m:15s
E155_Rep1	32m:16s	8m:9s	7m:37s	8m:55s
E155_Rep2	30m:25s	8m:9s	8m:21s	7m:5s
E165_Rep1	42m:0s	8m:52s	9m:13s	10m:55s
E165_Rep2	38m:35s	8m:8s	8m:18s	8m:49s
E175_Rep1	2h:17m:9s	10m:37s	7m:49s	13m:31s
E175_Rep2	3h:16m:55s	10m:32s	7m:38s	13m:53s
P0_Rep1	3h:39m:45s	10m:41s	8m:6s	11m:44s
P0_Rep2	3h:52m:16s	10m:38s	7m:46s	12m:15s
DLPFC 151673	6h:34min:18s	25m:27s	19m:19s	20m:15s
Adult mouse brain (from Stereo-seq)	/	31h:15m:13s	12m:52s	30h:7m:42s

Supplementary table 5. Gene lists for SVG c1, c2 and c3. The genes with obvious expressions in other regions were removed. In SVG c3, we did not strictly distinguish ventricle-specific and hippocampus-specific SVGs due to relatively low spatial resolution and data variation in 10X Visium.

SVG cluster	Gene list
SVG c1	*3110039M20Rik, Bcl11b, Bhlhe22, Fezf2, FoxG1, Hivep2, Mpped1, Neurod2, Neurod6, Tbr1, Zbtb18, Zeb2*
SVG c2	*9130024F11Rik, Mef2c, Satb2, Sla, Sox5*
SVG c3	*Eomes, Fbln2, Gas1, Gm11266, Insm1, Mki67, Neurod1, Neurog2, Rlbp1, Sema3c, Sstr2, Tac2, Thrsp, Top2a, Zbtb20*

Supplementary table 6. Primary antibody information.

Antibody	Company	Number	Origin
Tbr1	Abcam	ab183032	Rabbit
Satb2	Abcam	ab92446	Rabbit
Zbtb20	Abcam	ab243143	Rat
Nr4a2	R&D	AF2156-SP	Goat
Sox2	Santa Cruz	sc-365823	Mouse
Calb2	CST	#92635	Rabbit
NeuroD1	CST	#4373S	Rabbit
Nefm	Abcam	ab254348	Rabbit
